# Extracellular vesicle therapy in neurological disorders

**DOI:** 10.1186/s12929-024-01075-w

**Published:** 2024-08-25

**Authors:** Napasiri Putthanbut, Jea Young Lee, Cesario V. Borlongan

**Affiliations:** 1 Department of Neurosurgery, Center of Aging and Brain Repair, University of South Florida, Tampa, USA; 2 Department of Medicine, Faculty of Medicine Siriraj Hospital, Mahidol University, Salaya, Thailand

**Keywords:** Extracellular vesicle therapy, EVs, Neurological disorders, Regenerative therapy

## Abstract

Extracellular vesicles (EVs) are vital for cell-to-cell communication, transferring proteins, lipids, and nucleic acids in various physiological and pathological processes. They play crucial roles in immune modulation and tissue regeneration but are also involved in pathogenic conditions like inflammation and degenerative disorders. EVs have heterogeneous populations and cargo, with numerous subpopulations currently under investigations. EV therapy shows promise in stimulating tissue repair and serving as a drug delivery vehicle, offering advantages over cell therapy, such as ease of engineering and minimal risk of tumorigenesis. However, challenges remain, including inconsistent nomenclature, complex characterization, and underdeveloped large-scale production protocols. This review highlights the recent advances and significance of EVs heterogeneity, emphasizing the need for a better understanding of their roles in disease pathologies to develop tailored EV therapies for clinical applications in neurological disorders.

**Graphical abstract:**

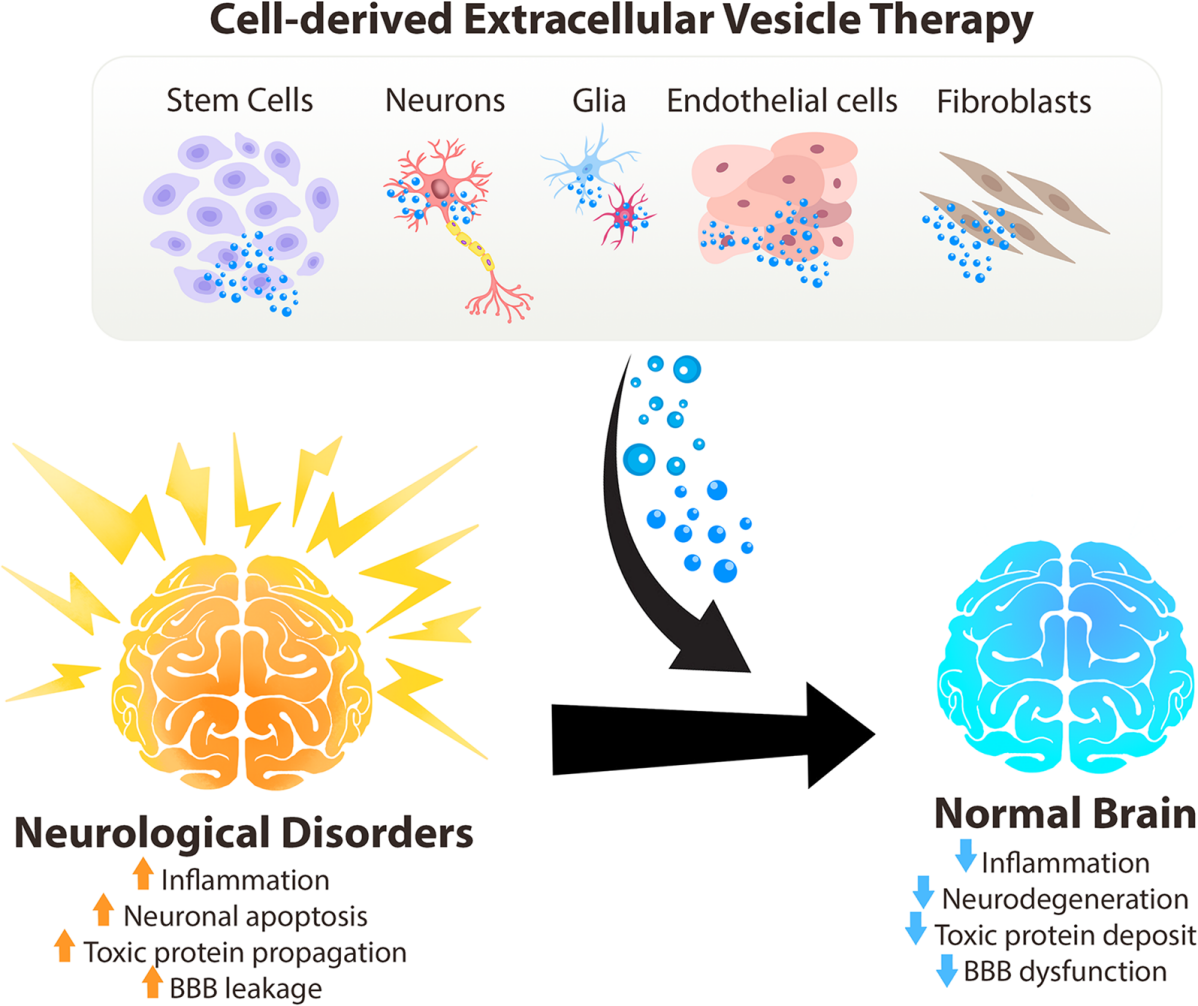

## Highlights

This paper reviews recent advances in EV subpopulation and characterization. We discuss the potential of EVs to address multiple aspects of neurological diseases, including neuroinflammation, mitochondrial dysfunction, apoptosis, and blood-brain barrier leakage. The review emphasizes the complexity and heterogeneity of EVs, highlighting the need for better characterization and classification to optimize therapeutic applications. By understanding EV subtypes and their roles, we can develop more effective and tailored EV therapies for clinical use in treating neurological conditions.

## Introduction

Extracellular vesicles (EVs) play a vital role in cell-to-cell communication, facilitating the transfer of proteins, lipids, and nucleic acids across various physiological and pathological processes acids [1, 2]. EVs were previously categorized into three classes based on their biogenesis: exosomes, microvesicles (MVs), and apoptotic bodies [1]. While numerous subpopulations continue to be identified, a clear understanding of their distinct functions remains elusive. Some aspects of biogenesis and regulation overlap among these classes. Furthermore, the heterogeneity of EV populations and their cargo is influenced by various factors [3]. Thus, in this study, we opt to classify EVs based on their size, providing a more generalized framework while also leaving room for future investigations.

Since EVs play pivotal roles in immune modulation [4] and tissue regeneration [5, 6], EVs represent a promising avenue for therapy across various medical domains. Compared to cell therapy, EVs offer several advantages, including greater versatility in delivery routes, ease of engineering, more concentrated cargo, absence of ethical concerns, and minimal risk of tumorigenesis or alloimmunization [7]. Moreover, EVs serve as a promising drug delivery platform [8–10]. However, several limitations hinder the real-world application of EVs, especially the characterization of their heterogenicity and exact therapeutic mechanisms.

This review underscores the importance of studying EV heterogeneity for therapeutic purposes in neurological disorders. Initially, we will delineate the various classes of EVs and elucidate the factors influencing their heterogeneity. Subsequent sections will delve into studies concerning the clinical utilization of EVs across diverse neurological disorders (Fig. 1). A better understanding of EV subpopulations and functions will pave the way for more tailored EV therapies.

### Extracellular vesicle subtypes

EVs are lipid bilayer-bound vesicles released by cells, varying from 30 nm to 2000 nm in diameter, and cannot replicate [1, 11]. Various classification systems exist for EVs, including their cellular origin, biological function, or biogenesis (Table 1). However, there is still not yet a consensus on EV classification. Although distinct mechanisms underlie the formation of each type of EV, there are notable overlaps between subpopulations. For example, all EV classes involve actin-myosin interactions [29–31] and translocation of phosphatidylserine [32, 33]. A combination of markers is commonly utilized to define EV subtypes [34].

**Table 1:** EV populations and subpopulations

Class	EV types	Size	Marker	Biogenesis	References
Small EVs (< 200 nm)	Exosomes	30–150 nm	CD63, Syntenin, LAMP1/2, ALIX, TSG101, CD9, CD81	Multivesicular bodies	[12–14]
	Small ectosomes	30–150 nm	CD147, CD9, CD81	Plasma membrane budding	[15]
	Protrusion-derived ectosomes	30 nm	Cholesterol, HSP90, cytoskeleton, prominin-1 (CD133)	Plasma membrane budding	[16]
	ARMMs	45–100 nm	TSG101, ARRDC1, VSP4 ATPase	Plasma membrane budding	[17, 18]
	Intracellular membrane-derived ectosomes	50–120 nm	Negatively charged phospholipid, cytokines	Plasma membrane budding (fast releasing method)	[19]
Small to large EVs	MVs	50–1000 nm	Annexin A1, annexin A2, a-actinin 4, ARF6, VCAMP3	Plasma membrane budding	[14, 20, 21]
	Apoptotic bodies	40–4,000 nm	Annexin V, TSP, C3b ICAM-3, phosphatidylserine, histone, mitochondrial content	Apoptosis	[14]
Large EVs (> 200 nm)	Large oncosomes	1–10 μm	Cytokeratin 18, caveolin-1, ARF6, GAPDH, HSPA5, V-ATPase G1, Annexin A1	Plasma membrane budding	[14, 22]
	Migrasomes	500–3000 nm	TSPAN4, cholesterol, integrin a5	Migration fiber	[23–25]
	Midbody remnants	200–600 nm	Microtubules, MKLP1, RACGAP1	Cytokinesis	[26]
	Exopheres	3.5–4 μm	Phosphatidylserine, LC3, Huntingtin, Tau, Annexin V, damaged mitochondria, Mitochondrial content	Unknown, Autophagy-related	[27, 28]

Numerous factors contribute to the heterogeneity of EVs, including cellular source, physiological state, and biological environment. EVs isolated from mesenchymal stem cells (MSCs) originating from different tissues exhibit variations in composition and function [35]. EVs from higher passage MSCs demonstrate reduced efficacy compared to those from younger cells [36]. EVs secreted from basolateral epithelial cells utilize distinct pathways compared to those from apical cells [20, 37]. A myriad of cell culture paradigms, such as 2D and 3D scaffolds, also impact EV composition [38, 39]. Furthermore, external stimuli such as inflammatory signals, ATP, heat stress, intracellular calcium levels, hypoxia, and various others can alter the composition of EVs, leading to environmental modification in EV therapy optimization [40–43].

Due to the difficulty in EV characterization, EVs can be roughly divided into small (< 200 nm) and large (> 200 nm) sizes. Small EVs, such as exosomes and MVs, are generally found to be more therapeutic than large EVs. However, the mechanism of action of each class of small EVs is different. For example, exosomes carry anti-inflammatory microRNA (miRNA) and growth factor receptors while MVs transfer functional mitochondria. Small EVs are also frequently used as drug delivery vehicles more often than large EVs. Finding the optimal treatment regimens of EVs and MVs will advance their safe and effective therapeutic applications for neurological disorders.

### Small EVs

Small EVs are the most studied group of EVs, especially exosomes and MVs. Exosomes arise via the inward budding of multivesicular bodies (MVBs), generating intraluminal vesicles (ILVs) [44]. MVBs may fuse with the cell membrane for exosome secretion or with lysosomes for degradation [45, 46]. There are different exosome subtypes, as indicated by variations in ILV formation and cargo-loading mechanisms [2]. Exosomes employ two primary cargo sorting mechanisms: the endosomal sorting complexes required for transport (ESCRT) pathway and the ceramide-dependent mechanism [47, 48]. Key regulators of the ESCRT pathway, such as programmed cell death 6-interacting protein (PDCD6IP or ALIX) and tumor susceptibility gene 101 protein (TSG101), are often used as exosome markers [49, 50]. Tetraspanins, including CD9, CD63, CD81, and CD82, also play a role in cargo selection [51, 52]. Other well-known markers for exosomes are LAMP1/2 and Syntenin [15].

Small ectosomes are released through plasma membrane budding [15]. Differentiating between small ectosomes and exosomes within the small EV population requires a combination of presenting plasma membrane molecules and lacking endosomal markers, such as CD63. Small ectosomes, albeit similar in size to exosomes, are more enriched in centrosomal, ribosomal, and mitochondrial proteins and contain fewer oncogenic genes compared to exosomes [53].

Protrusion-derived ectosomes are released from membrane protrusions such as filopodia, microvilli, and cilia during cellular movement [16]. Bin–Amphiphysin–Rvs (I-BAR) domain-containing proteins, including MIM and IRSp53, connect the plasma membrane to actin, GTPase, and phosphoinositides [54, 55] to facilitate protrusion forming and also ectosome release. CD133, also known as prominin, is necessary for the release of ectosomes from microvilli [56, 57]. There are three proposed scission processes: first, ESCRT machinery recruitment, as evidenced by viral-induced vesicle release [58]; second, actomyosin contractility causing plasma membrane scission through GTPase ADP-ribosylation factor, though its exact mechanism—whether mechanical contraction or molecular signaling—remains unclear; and third, shear friction from extracellular fluid flow contributing to ectosome release. Prominin-1 and I-BAR domain-containing proteins are potential markers for protrusion-derived ectosomes, but further research is needed for precise classification.

Arrestin domain-containing protein 1 (ARRDC1)-mediated microvesicles (ARMMs) are generated through plasma membrane budding, akin to virus-induced MVs. The PSAP motif of ARRDC1 on the plasma membrane recruits endosomal TSG101 to the cell membrane for budding. The VPS4 ATPase facilitates the final budding process of ARMMs, similar to exosome and viral budding [17]. ARRDC1 is specifically recruited in PPXY-mediated budding and interacts with HECT ubiquitin ligases such as WW1, WW2, and [18]. ARRDC1 serves as a marker for ARMMs, and due to their biogenesis, these MVs are negative for endosomal markers such as LAMP3 and CD63 [17].

Intracellular membrane-derived ectosomes exhibit two distinct release mechanisms: slow-releasing and fast-releasing. In the slow-releasing method, ectosomes are secreted via outward budding of the plasma membrane [19]. Conversely, the fast-releasing process involves intracellular vesicles being directly squeezed out through pores in the plasma membrane, resulting in ectosomes with different components compared to conventional plasma membrane-derived ectosomes. Ectosomes released in the fast phase possess negatively charged phospholipids, typically found in the inner membrane, whereas conventional ectosomes share similar plasma membrane components with the host cells.

Small EVs, particularly exosomes and small ectosomes, have been extensively studied for their critical roles in intercellular communication. Despite differences in their biogenesis, overlapping mechanisms highlight the necessity for combination markers in their characterization. However, the characterization of their subpopulation within the same classes is still not clear. The heterogenicity of their cargo leads to inconsistent results in treatment outcomes and mechanism of action. Better characterization and isolation techniques as well as a complete content profile are important for their utilization. Protrusion-derived ectosomes and ARMMs, though less understood, offer insights into cell movement-related and viral-induced communication, respectively. Intracellular membrane-derived ectosomes represent another subtype, and the impact of their negatively charged membranes on targeting ability remains unexplored. Although exosomes and small ectosomes from sources like stem cells have shown therapeutic potential, their optimal treatment regimen, as well as the utility of yet to be examined small EVs, remain unclear and warrant additional studies.

### Small-to-large EVs

MVs, generally known as ectosomes or shedding vesicles, are generated via direct outward budding of the cell membrane [46, 59]. Despite ongoing research, the mechanisms underlying cargo sorting in MVs remain elusive, with various proposed pathways such as calcium-induced cytoskeletal remodeling [60, 61], protein kinase C and purinergic receptors P2X7/P2Y [62, 63]. ARF6, TSG101, ceramide, and lipid rafts regulate the formation of both MVs and exosomes, suggesting shared underlying mechanisms [64, 65]. Following shedding, MVs either degrade rapidly, releasing their contents into the extracellular space, or engage in communication with specific target cells through receptor signaling, direct fusion with the plasma membrane, or endocytosis [66].

Apoptotic bodies, also known as apoptosomes, are vesicles released during programmed cell death [34, 67]. These bodies typically exhibit a large size and contain organelles within their vesicular structure [68, 69]. Apoptotic bodies are expelled via a 'beads-on-a-string' formation, a screening procedure that selectively excludes nuclear content from these bodies [70, 71]. Concurrently, smaller vesicles are also released, potentially originating from membrane blebbing during apoptosis [70, 72]. Most apoptotic bodies are phagocytosed by local macrophages, which recognize them through Annexin V, thrombospondin, and C3b [73–75]. Apoptotic bodies also carry genetic cargo, potentially involved in tumor metastasis [76, 77].

Small-to-large EVs encompass a wide range of sizes and properties. MVs exemplify a pivotal class of EV that has garnered much interest. Initially, MVs were often confused with exosomes due to their similar size. However, discoveries in their distinct biogenesis, cargo, and markers have clarified the diversity of EVs. MVs deserve more attention due to varying reports on their therapeutic effects. Their larger size and direct membrane budding biogenesis may be advantageous for developing drug delivery vehicles, as MVs can carry more content and functional organelles such as mitochondria. On the other hand, apoptotic bodies are generally pathogenic. Enhancing the clearance of apoptotic bodies or inhibiting their uptake by peripheral tissues can be a promising area of study to suppress inflammation and tumor metastasis, especially in relation to brain function will advance EV use in neurological disorders.

### Large EVs

Large oncosomes manifest as large ectosomes originating from tumor cells. Oncoproteins such as MyrAkt1, HB-EGF, and caveolin-1 (Cav-1), as well as EGFR overexpression resulting in plasma membrane blebbing. The cargo of oncosomes can induce tumor spreading and progression [22]. In prostate cancer, tumor cells release oncosomes containing AKT1 kinase. The internalization of oncosomes leads to fibroblast reprogramming, promoting tumor growth via MYC activation and environmental modulation. Inhibition of oncosome uptake can prevent tumor progression, offering a novel therapeutic approach for cancer [78]. In addition to oncoproteins, cytokeratin 18 (CK18) is proposed as a marker for oncosomes.

Migrasomes are formed during cell migration [23]. During this process, large vesicles develop at the tips of retracting fibers behind the cells, relying on actin polymerization. Migrasomes contain abundant small vesicles, with diameters ranging from 50 to 100 nm, resembling pomegranates. Tetraspanin-4 (TSPAN4) has been identified as the most prominent marker for migrasomes, along with TSPAN7, cholesterol, and integrin α5 [24, 25]. Although the precise function of migrasomes remains unclear, they are hypothesized to facilitate cell–cell communication in a specific direction related to cell migration [23].

Midbody remnants, another type of ectosome, are remnants of the intercellular bridge formed during cell division [26]. They are rich in cytoskeletal proteins such as microtubules, centralspindlin, and the chromosomal passenger complex. This structure can either retract into daughter cells or be released into the extracellular space, where they may be degraded or internalized by neighboring cells. Midbody remnants are primarily reported in cancer cells [26, 79]. Uptake of midbody remnants secreted from cancer cells can induce a malignant phenotype in fibroblasts [26]. Midbody derivatives selectively accumulate in stem cells, leading to loss of differentiation and autophagy evasion through the binding of the CEP55 midbody protein to the autophagic receptor NBR1 [80]. The precise mechanism of action, whether through mutated protein cargo or epigenetic dysregulation, remains unknown.

Exophers represent large ectosomes with ambiguous biogenesis, containing organelles, particularly mitochondria and lysosomes, as well as protein aggregates such as huntingtin and tau. Most of the secreted exophers are taken up by neighboring cells. Exophers have also been found in remote tissues, suggesting secondary release after uptake [27]. During stress, cardiomyocytes excrete dysfunctional mitochondria into exophers driven by autophagy machinery. Impaired autophagy causes the accumulation of anomalous mitochondria, leading to dysfunctional ventricles and metabolism [81]. The role of exophers may be the eradication of toxins and dysfunctional organelles during stress.

Taken together, large oncosomes, migrasomes, midbody remnants, and exophers represent diverse and specialized ectosomes with significant roles in cellular processes and disease progression. Large oncosomes and midbody remnants, which play crucial roles in tumor progression and spreading, are potential targets for cancer therapy through the inhibition of their biogenesis and uptake. The discovery of migrasomes suggests direction-specific communication, but more research is needed to understand their effects and control mechanisms fully. Exophers help alleviate cellular stress by removing dysfunctional components. Understanding their function will elucidate organelle transfer and cellular stress management. Enhancing exopher production in situations involving organelle dysfunction-related diseases may mitigate pathogenesis. Probing the pathological and treatment modalities of these large EVs presents new avenues for research and therapeutic development for brain diseases.

### EV therapy in neurological disorders

EVs play crucial roles in disease pathogenesis, especially immunomodulation (Table 2). Inflammation is involved across a spectrum of diseases, including degeneration, cancer, infections, and trauma [112]. EV therapy can be categorized into two methods: inhibiting pathogenic EVs and promoting therapeutic EVs [1].

**Table 2:** Pathologic and therapeutic effects of EVs

EVs	Effects	References
Pathogenic EVs	Prion-like misfolded protein propagation, ex. tau, α-synuclein, mHTT	[82–85]
	Proinflammatory miRNA and protein transfer	[86, 87]
	Promote inflammatory M1 microglia and A1 astrocytes polarization	[88]
	Cellular proliferation ex. tumorigenesis	[89]
	Hypercoagulation	[90]
Therapeutic EVs	Anti-inflammatory miRNA and protein transfer	[1, 91–94]
	Promote anti-inflammatory M2 microglia and A2 astrocytes phenotype change	[93]
	Anti-apoptosis miRNA and protein transfer	[95–99]
	Recover mitochondria function	[100, 101]
	Reduce endoplasmic reticulum stress	[102]
	Neurogenesis, neurite outgrowth, and remyelination	[103, 104]
	Increase angiogenesis	[105–109]
	Restore BBB integrity	[110]
	Restore normal microbiome-gut-brain axis	[111]

Pathologic EVs carry pro-inflammatory factors and toxic proteins. Blocking phosphatidylserine, a surface component crucial for EV sorting and uptake, can reduce EV uptake, consequently diminishing tumor growth and angiogenesis [113]. Additionally, targeting FAS ligands on EVs with anti-FASL monoclonal antibodies has been shown to reduce tumor progression [114]. However, inhibiting the EV cascade lacks specificity and may disrupt physiological processes.

Therapeutic EVs suppress inflammation and promote tissue regeneration. Therapeutic EVs can be derived from various sources, with stem cells being the most extensively studied due to their versatility [1]. While stem cell transplantation has shown great results in treating neurological disorders, their mechanism of action is primarily through paracrine effects rather than cellular replacement [115, 116]. Culture media derived from healthy cells can alleviate inflammation, promote angiogenesis, and restore function in the same way as cell transplantation [91]. EVs mediating these paracrine effects contain miRNA, non-coding RNA, growth factors, receptors, proteins, and lipids [1]. miRNA is believed to be indispensable for the therapeutic effects. EVs contain components involved in RNA transportation and processing such as RNA-binding proteins Staufen homolog 1 (STAU1), STAU2, Argonaute 2 (AGO2), and trinucleotide repeat-containing gene 6A protein (TNRC6A; also known as GW182). Ago2 knockdown diminishes the therapeutic effects of MSC-EVs [117].

Neurological disorders are always notoriously challenging due to the limited regenerative capabilities of neurons and the selective blood–brain barrier (BBB) preventing CNS entry of many therapeutic agents. EV therapy has a great advantage as it can precisely target brain parenchyma and effectively cross BBB (Table 3). Systemic administration or invasive methods such as intranasal spray can deliver EVs into the central nervous system (CNS). Here we summarize advances of EV therapy in neurological disorders across various pathologies to give a complete view of EV application: ischemia, trauma, degeneration, autoimmune-induced inflammation, and genetic mutation. In each disease section, we cover pathogenesis, pathologic EV involvement, mechanisms of therapeutic action, and EV optimization.

**Table 3:** Cell-derived EV therapy in neurological disorders

Disease	EVs sources	Outcomes	Mechanisms	Type/references
Ischemic stroke	MSCs	↓Astrocyte apoptosis ↓Inflammatory marker in astrocyte ↓Oligodendrocyte apoptosis	miR-138-5p downregulates lipocalin 2 (LCN2) miR-134 suppresses caspase-8	In vitro: [95, 98]
	ASCs	↓Infarct size ↑Neurological recovery ↑Angiogensis ↓Inflammatory, ROS, apoptotic, and fibrosis, BBB leakage	MALAT1 recruits splice factor serine-arginine-rich splice factor 2 (SRSF2) → ↑splicing of PKCδII → ↑neuron proliferation miR-181b-5p targets transient receptor potential melastatin 7 (TRPM7) → ↑endothelial cell migration miR-126 mediates neuroprotection	In vitro: [97, 106] In vivo: [105, 108]
	Neurons	↑BBB integrity	miR-132 upregulate eef2k → ↑VE-cadherin	In vitro: [109]
	Endothelial cells	↑Mitochondrial function ↑Neurological outcomes ↓Infarct sizes ↓Apoptosis	Mitochondrial component transfer in medium-to-large EVs miR-199a-5p suppresses ER stress miR-126 mediates neurorestoration	In vitro: [96, 101] In vivo: [101, 102, 107]
	Microglia	↑Angiogensis	miRNA-26a mediates angiogensis	In vitro: [109] In vivo: [109]
	Serum	↑Synaptic transmission/plasticity, ↑Spatial learning and memory	↓Cyclooxygenase-2 (COX-2) expression	In vivo: [118]
Hemorrhagic stroke	MSCs	↑Hematoma clearance ↓Brain edema ↓Neuronal apoptosis ↑Neurological function ↑Regulatory T cells ↑M2 polarization	Blocking CD47- signal regulatory protein alpha (SIRPα) interactions Activation of the BDNF/TrkB/CREB signaling pathway Inhibited NF-κB and activated AMPK signaling pathways Decreased transcription of high-mobility group box 1 protein (HMGB1) and miRNA129-5p miR-140-5p targets and downregulates ALK5 and NOX2 expression	In vivo: [119–123]
	ASCs	↑Neurological function ↓Neuron loss	miR-19b-3p-modified ADSCs inhibit ferroptosis	In vitro: [124] In vivo: [124]
	NSCs	↑Behavioral recovery ↑Angiogenesis	Akt1, GDNF, and BDNF overexpressions increase resistance to oxidative stress and promote neuroprotection	In vivo: [125–127]
Traumatic brain injury	MSCs	↑Pattern separation and spatial learning ↓Neuroinflammation ↑M2 microglial polarization ↑Hippocampal neurogenesis ↑Synaptogenesis and neuroplasticity	miR-140-5p modulates HDAC7/AKAP12/cAMP/PKA/CREB pathway Enhancing the BDNF-ERK-CREB signaling pathway Inhibit NLRP3 inflammasome and p38/MAPK signaling pathways	In vivo: [99, 104, 128–130]
	Endothelial progenitor cells	↑BBB integrity	Inhibits PTEN/AKT signaling pathway	In vitro: [131] In vivo: [107, 131]
	Astrocytes	↑M2 microglia transformation ↑Neurological outcomes	miR-873a-5p inhibits ERK/NF-κB pathway	In vivo: [93]
	Microglia	↑M2 microglia transformation ↑Neurological outcomes	miR-124-3p inhibits TLR4 pathway, autophagy-associated FIP200 gene, and Rela/ApoE pathway	In vitro: [132] In vivo: [132–135]
Spinal cord injury	MSCs	↑Neuronal proliferation ↓Apoptosis ↓Inflammation ↓Lesion size ↑Motor function ↑A2 astrocytes	Activation of Wnt/β-catenin signaling pathway miR-21 targets the JAK2/STAT3 signaling pathway in astrocyte phenotypic alterations miR-211-5p downregulates COX2 mRNA miR-21a-5p blocks PELI1 expression → ↓pyroptosis, ↑autophagy miR-125a-3p inhibits NET formation miR-26b-5p targets KDM6A → ↑H3K27me3 → ↓NOX4 → ↓ROS	In vitro: [136, 137] In vivo: [94, 136–139]
	NPSCs	↓Inflammation ↓Apoptosis ↑Motor function ↑Angiogenesis	14-3-3t protein interacts with Beclin-1 to ↑autophagy NLRP3 inflammasome formation inhibition VEGF promote angiogenesis	In vivo: [140–142]
	Neurons	↓M1 microglia and A1 astrocytes	miR-124-3p/MYH9 axis interacts with PI3K/AKT/NF-κB signaling pathway	In vivo: [143]
Peripheral nerve injury	MSCs	↑Axonal regeneration ↑Motor function ↓Inflammation	cyclin Ki67	In vitro: [3] In vivo: [144–146]
	ASCs	↑Axonal regeneration		In vivo: [144]
	SCs	↑Axonal regeneration	GTPase RhoA inhibition miRNA-21 ↓PTEN and ↑PI3-kinase pathway in neuron proliferation	In vitro: [147, 148] In vivo: [148]
	Neurons	↑Axonal regeneration		In vitro: [3]
	Macrophages	↑SC proliferation ↑Nerve growth factors	miR-223 increases NGF and Laminin	In vitro: [149] In vivo: [149]
	OECs	↑Axonal regeneration and myelination	↑PI3K/Akt signaling pathway ↓JNK signaling pathway	In vivo: [150]
	Pericytes	↑Angiogenesis ↑Nerve regeneration ↑BDNF, neurotrophin-3, and NGF		In vivo: [151]
	Dental pulp stem cells	↑Myelination	miR-122-5p inhibits P53-mediated autophagy	In vitro: [152] In vivo: [152]
Epilepsy	MSCs	↓Neuron loss ↓Inflammation ↑Hippocampus neurogenesis ↑Cognitive and memory function		In vivo: [153]
Alzheimer’s disease	MSCs	↑Memory and cognitive function ↓Inflammation and oxidative stress ↑Neuroplasticity ↑Mitochondrial function	Catalase-mediated protection against ROS Nrf2 signaling pathway miR-146a inhibit NF-κB signaling miR-223 targets PTEN-PI3K/Akt pathway	In vitro: [154–158] In vivo: [155, 158–160]
	ASCs	↓Neuronal damages and apoptosis ↑Mitochondrial function		In vitro: [161, 162] In vivo: [163]
	NSCs	↑Mitochondrial function ↑SIRT1 activation ↑Synaptic activity ↓Inflammation and oxidative stress ↓Cognitive deficits		In vivo: [164–166]
	Neuron	↓ Aβ deposit ↑Neuroplasticity	Aβ binding by EVs surface proteins such as prion proteins and GSLs	In vivo: [167, 168]
	CSF	↑Electrophysiological activity ↑Neurogenesis		In vivo: [167]
	HBMVECs	↑Aβ clearance ↑Cognitive function	P-glycoprotein on exosomes as an extracorporeal Aβ cleansing system	In vivo: [169]
Parkinson’s disease	MSCs	↓Apoptosis ↓Motor deficit ↓Dopaminergic neuron loss	Increase autophagy	In vitro: [170] In vivo: [170]
	SHEDs	↑Motor function ↑Tyrosine hydroxylase in striatum and substantia nigra ↓Apoptosis	Cu/Zn SOD1, TXN and PRDX6 proteins as antioxidants HSP70 gene transfer	In vivo: [171]
	Astrocyte	↓Cell death with ↓MKK4	miR-200a-3p down-regulates MKK4	In vitro: [172]
Amyotrophic lateral sclerosis	MSCs	↑BBB integrity		In vitro: [173, 174] In vivo: [173]
	ASCs	↑Motor function ↓Lumbar motoneuron loss ↓Gliosis ↑Mitochondrial function		In vitro: [175, 176] In vivo: [177]
Multiple sclerosis	MSCs	↓Neurological deficits ↓Inflammation and demyelination ↑M2 microglia		In vivo: [178, 179]
	Periodontal ligament stem cells	↓Inflammation ↓Apoptosis (STAT1, p53, caspase 3, and Bax)	CD90 induces IL-10 production	In vivo: [180]
	Microglia	↑Oligodendrocyte progenitor cells recruitment and differentiation	Lipid cargo enhances OPC maturation EV-associated S1P in stimulating OPC migration Astrocyte may be effector in oligotoxic cell maturation	In vitro: [181] In vivo: [181]
Huntington’s disease	ASCs	↑Mitochondrial function ↓N-terminal cleavaged mHTT ↓Apoptosis		In vitro: [182] In vivo: [183]
	NPSCs	↓N-Terminal Cleavaged Mhtt ↓Apoptosis		In vitro: [184]
	Fibroblast	↑GABAergic synapses and transmission		In vitro: [185–187]
	Blood serum	↓mHTT aggregation ↓Neuronal death ↓Inflammation and gliosis ↑Neuromuscular function		In vivo: [188]

### Stroke

Stroke, including ischemic and hemorrhagic strokes, ranks second as a cause of death and disability worldwide [189, 190]. The pathogenesis of brain injury following ischemia involves oxidative stress, inflammation, excitotoxicity, and apoptosis [191]. On the other hand, hemorrhagic stroke injury is from hematoma compression and increased intracranial pressure, subsequently also causing inflammation, excitotoxicity, and impaired BBB [192]. Interestingly, the upregulation of CD63 exosomes closely approximates with endogenous neurovascular unit regenerative process [193]. Although stroke intervention methods like mechanical thrombectomy and surgical decompression have rapidly improved, few treatments effectively address neuronal death [194]. Novel anti-inflammatory therapies targeting inflammatory cell recruitment have failed in clinical trials, suggesting that additional modalities, such as regeneration, may be necessary.

EV therapy can address multiple aspects of stroke pathophysiology and improve neurological outcomes [195], while minimizing complications associated with cell-based therapy [196]. Overall, most EV therapies improve infarct size, hematoma clearance, brain edema, and neurological functions [108, 119, 120, 124, 134]. Mechanistically, EVs derived from MSCs, adipose-derived stem cells (ASCs), and astrocytes promote anti-inflammatory M2 microglia polarization, suppress inflammatory cytokines, and reduce oxidative stress [88, 197, 198]. EVs carrying miR-132 can suppress eukaryotic elongation factor 2 kinase (eef2k) and restore VE-cadherin, an endothelial adhesive junction component [110]. ASCs-EVs also decrease aquaporin-4 (AQP-4) levels [105]. Mitochondria transfer by MVs and mitochondrial DNA via exosomes also increase the integrity of brain endothelial cells (BECs) [100, 101]. EVs effectively reduce apoptosis of neurons, oligodendrocytes, and astrocytes. EVs containing miR-138-5p reduce Lipocalin 2 and metastasis-associated lung adenocarcinoma transcript 1 (MALAT1), downregulating bax, caspase-3, caspase-8, and inflammatory cytokines while upregulating Bcl-2 and Cyclin family proteins [95, 98]. EVs containing miR-199a-5p also reduce apoptosis by ameliorating endoplasmic reticulum stress [102]. EVs promote neurogenesis and angiogenesis through miR-126, miR-26a, miR-181b, and multiple growth factors [107–109]. They also benefit the microbiome-gut-brain axis damaged after ischemic stroke by downregulating Interleukin (IL) 17 and upregulating IL-10, which modulates microbiota diversity and intestinal immunity [111].

Preconditioning, source selection, and combination therapy improve EV therapy efficacy [199, 200]. Pretreatment with hypoxia in MSC restores the BBB more effectively [201]. EVs from macrophages and microglia pretreated with IL-4 ameliorate apoptosis and promote angiogenesis, while EVs from tumor necrosis factor alpha (TNF-α) pretreated endothelial progenitor cells have the opposite effect [202, 203]. Mitochondria-containing EVs derived from brain endothelial cells from the same species donor are more effective in mitochondria transfer [204]. Better outcomes are observed in combined therapies with exercise, enriched environments, acupuncture, brain stimulation, and hypothermia [199].

EV therapy for stroke offers multiple therapeutic benefits, including anti-neuroinflammation, anti-apoptosis, BBB restoration, neurogenesis, and angiogenesis. However, the diverse range of therapeutic cargo and molecular pathways reported, even from the same cell source, leads to confusion about which cargo acts as the primary regulator. It remains unclear whether these components coexist within the same vesicles or are distributed among different EV subtypes. Various cell sources have been studied, but their effects on different domains of pathogenesis have not yet been compared. For instance, EVs derived from stem cells and anti-inflammatory glia may primarily modulate inflammation, while those from endothelial cells may be more effective in promoting angiogenesis. Preconditioning parent cells with hypoxia, which mimics ischemic stroke conditions, induces the secretion of EVs suited for such situations. Given that neuronal death is a primary cause of disabilities in ischemic stroke, EV therapy should focus more on long-term neurogenesis and functional recovery. Moreover, while most preclinical studies deliver EVs within the first hour following the lesion induction, actual patients typically receive treatment at a much later stage, implying the need to modify the experiment design [205]. Finally, the safety profile of EVs is a concern. Overexpression of vascular endothelial growth factor (VEGF) and hypoxia-inducible factor-1 (HIF-1) can potentially lead to BBB leakage and brain edema [206, 207]. Therefore, recognizing the risks and benefits of these secreted growth factors should guide the timing of administration and expression of specific EV components and will require further investigations.

### Traumatic brain injury (TBI)

TBI is the most common cause of morbidity and mortality in the young population, commonly resulting from falls and traffic accidents [208]. Neurological damage occurs both from the initial impact (primary brain injury) and subsequent ischemia due to brain swelling (secondary injury). Despite several neuroprotective strategies, such as antioxidants, *N*-methyl-d-aspartate (NMDA) receptor antagonists, and calcium channel blockers, there is only minimal improvement [209]. TBI brain-derived EVs induce multisystemic organ dysfunction. Lactadherin can eliminate brain-derived EVs and improve coagulopathy and inflammation [210–212].

Like ischemic stroke, therapeutic EVs modulate neuroinflammation by promoting M2 microglia polarization and reducing pro-inflammatory cytokines, subsequently decreasing neuronal apoptosis. Astrocytes-derived EVs carry miR-873a-5p, inhibiting the ERK/NF-κB signaling pathway [93]. Additionally, microglia-derived EVs contain miR-124–3p, which suppress mTOR signaling, autophagy-associated FIP200 gene, Rela/ApoE pathway, and toll-like receptor-4 (TLR4) signaling pathway [133–135]. MSC-EVs suppress TRAF6 in the TLR4 signaling pathway via miR-146a, the cAMP/PKA/CREB pathway via miR-140-5p, and the CysLT2R-ERK1/2 pathway mediating M1 polarization [128, 213–215]. MSC-EVs also decrease the pro-apoptotic factor Bax while increasing the anti-apoptotic factor Bcl-2 expression [99]. For long-term complications of TBI, MSC-EVs inhibit chronic activation of the NLRP3-p38/MAPK signaling pathway and improve long-term cognitive function [129]. EVs also increase neuron survival by stimulating myelination [103] and transferring neuroprotective agents such as Apo-lipoprotein D (ApoD) [216, 217]. EVs upregulate genes associated with neurogenesis, synaptogenesis, and neuroplasticity while downregulating non-neuronal differentiation genes [104]. Neural stem cell-derived EVs (NSC-EVs) increase neurogenesis through miR-320-5p, miR-210, miR-21a, and miR-9 [218–221].

EV therapy effectively addresses neuroinflammation in both the acute and chronic phases of TBI while also promoting neurogenesis. Various miRNAs and their associated pathways are integral to this mechanism. Tailoring specific EV properties to different phases of pathology can enhance treatment outcomes. For example, administering anti-inflammatory EVs in the hyperacute phase may prevent secondary brain injury, while using pro-neurogenesis and pro-angiogenesis EVs later can improve functional recovery. Besides therapeutic EVs, temporarily blocking pathological EVs that signal inflammation may also benefit patients by reducing secondary damage, though current techniques for blocking EV release, explored in models of neurodegenerative disorders, still lack precision. Furthermore, since TBI often coincides with systemic damage such as hemorrhagic shock or organ trauma, it is crucial to investigate the effects and systemic distribution of EVs in these contexts. The modification of EV sources in TBI has not been as thoroughly studied as in ischemic stroke, presenting an opportunity to apply existing stroke-relevant knowledge to this field.

### Spinal cord injury (SCI)

SCI, often caused by trauma, is a chronic disability that imposes a significant healthcare burden [222]. Like TBI, SCI involves primary injury from traction and compression forces, followed by secondary hypoperfusion due to spinal cord swelling [223, 224]. Inflammation further exacerbates the condition, leading to excitotoxicity and reactive oxygen species (ROS)-induced apoptosis. Despite therapeutic advances over the past decade, treatments for SCI, such as surgical decompression, corticosteroids, and neuroprotective agents, remain controversial and largely ineffective. Novel therapeutic strategies focus on limiting cell death, promoting regeneration, and restoring myelination. Pathologic EVs inhibit axon regeneration, induce systemic inflammation, and cause multi-organ damage [225].

Neural stem cell/progenitor cell (NPSC)-derived EVs reduce inflammation by suppressing the NLRP3 inflammasome and increasing autophagy-regulating Beclin-1 expression through the 14-3-3τ protein [140, 141]. They also induce angiogenesis by transferring VEGF to endothelial cells [142]. Cortical neuron-derived EVs suppress pro-inflammatory microglia and astrocytes through miR-124-3p [143]. MSC-derived EVs promote A1-to-A2 astrocyte conversion via miR-21 [136], suppress cyclooxygenase 2 (COX2) mRNA via miR-211-5p [94], inhibit macrophage/microglial pyroptosis through the miR-21a-5p/PELI1 axis-mediated autophagy pathway [226], and reduce neutrophil extracellular trap (NET) formation in both the spinal cord and circulation via miR-125a-3p [167]. Additionally, miR-26b-5p-enriched MSC-EVs epigenetically regulate the KDM6A/NOX4 axis to suppress inflammation and ROS production [139].

Environmentally modulated EVs perform better than naïve EVs [225]. Microglia-derived EVs function differently under pro-inflammatory or pro-regenerative preconditioning [227]. Similarly, EVs isolated from hypoxic MSCs have significantly higher potency in miR-146a-5p-mediated immune modulation [228]. NPSCs primed with insulin-like growth factor 1 (IGF-1) secrete EVs highly enriched in miR-219a-2-3p, which induce oligodendrocyte progenitor cell (OPC) maturation and promote axonal regeneration [229].

Similar to TBI, EVs help prevent secondary injury in SCI by suppressing inflammation. To establish EVs as a viable immunosuppressant therapy, comparative studies with conventional corticosteroids are needed to evaluate their efficacy. Currently, EV sources in SCI are limited to MSCs, NPSCs, and neurons. Exploring additional sources, such as endothelial cells and immune cells, can provide a more comprehensive treatment approach for SCI. Additionally, assessing the extent of SCI severity and determining the optimal treatment regimen specific to the EV source in tandem with adjunct therapies will be crucial for maximizing the beneficial effects of EVs.

### Peripheral nerve injury (PNI)

Despite advances in neurology, there is still no effective therapy for nerve regeneration [230]. Although axons can regrow after injury, the growth rate is extremely slow and often complicated by inflammation and scar formation [231]. Neurorrhaphy is feasible only for short-gap injuries, while autologous nerve grafts have limitations, including nerve source selection and donor site dysfunction. Nerve guide conduits and cell therapy are potential candidates for PNI treatment but still face several complications [230, 232]. Pathologic EVs play an important role in blocking nerve growth. Schwann cells (SCs) secrete miR-1, inhibiting brain-derived neurotrophic factor (BDNF) expression and blocking axonal regeneration. miR-1 inhibitors efficiently improve SC proliferation and migration [233]. Injured dorsal root ganglia (DRG) secrete miR-23a-enriched EVs, targeting the A20 gene and promoting M1 macrophage polarization. EV-miR-23a antagomir reduces M1 macrophages, pro-inflammatory cytokines, and pain hypersensitivity [234].

EVs effectively target injured neurons and peripheral axons [235]. SC-derived EVs are highly focused candidates for PNI treatment [230]. Following PNI, SCs dedifferentiate to a progenitor-like state, guiding axonal regeneration. Exosomes from dedifferentiated SCs significantly increase axonal growth by inhibiting GTPase RhoA [148] and downregulating PTEN by miR-21 [147]. MSC-exosomes enhance neurite outgrowth by expressing neural growth factors such as BDNF, fibroblast growth factor 1 (FGF-1), glial cell line-derived neurotrophic factor (GDNF), IGF-1, and nerve growth factor (NGF), while MSC-MVs have the opposite effect [3, 144]. However, MVs derived from M1 macrophages increase SC proliferation and migration compared to those from M0 macrophages [149]. Other beneficial cell sources for nerve growth include olfactory ensheathing cells (OECs), pericytes, dental pulp stem cells, and induced pluripotent stem cells (iPSCs) [150–152, 236, 237]. EVs also address complications of PNI, such as injury-induced neuropathic pain [235] and denervation-induced muscle atrophy [238]. EVs are also effective in treating non-traumatic peripheral neuropathy, such as diabetic peripheral neuropathy and chemotherapy-induced peripheral neuropathy [239, 240].

Multiple optimization techniques show promise in PNI treatment. Mechanical stimulation of SCs increases miR-23b-3p-enriched EVs, which promote DRG neuron survival and neurite outgrowth [241]. Platelet-rich plasma (PRP) supplementation upregulates c-Jun and GDNF in the EVs while also promoting parent cell viability [242, 243]. Hypoxic neural crest cells promote sensory neuron repair through miR-21-5p [244]. EVs combined with conduits offer more efficient PNI treatment [244–247]. Even more advanced, a superparamagnetic nanocomposite scaffold, which can mechanically stimulate encapsulated SCs to release EVs, optimizes noninvasive and remotely time-scheduled nerve repair [248].

PNI models demonstrate the potential of EV therapy in the peripheral nervous system (PNS). EVs facilitate nerve regeneration by restoring SCs and DRG neurons while simultaneously suppressing inflammation. However, different EV subtypes yield distinct outcomes: exosomes are therapeutic, whereas MVs can be pathological. Due to the size range overlap between exosomes and MVs, the purification and characterization of EVs are critical for effective treatment outcomes. In addition to conventional peripheral nerve models, exploring the effects of EV therapy on cranial nerve injuries is also needed. Advances in EV-conduit integration and noninvasive remote scheduling systems show great promise for the future of PNI treatment, offering more precise and effective therapeutic options.

### Epilepsy

Epilepsy stands as a major debilitating brain disorder complicated by numerous factors and genetic predispositions [249]. While antiepileptic medication can suppress seizures, it does not improve long-term outcomes. Epilepsy surgery is the most effective treatment but is only suitable for selected patients. Prolonged seizures, such as status epilepticus (SE), lead to an inflammatory cytokine storm mediated by activated microglia and reactive astrocytes, causing neurodegeneration, particularly in the hippocampus.

EVs can alleviate brain damage from epilepsy by modulating neuroinflammation [250], similar to stem cell transplantation [251]. However, there are only a few studies on EVs in epileptic models. Intranasal administration of MSC-exosomes immediately after SE reduces SE-induced injury in the hippocampus and preserves glutamatergic and GABAergic neurons [153]. Loading exogenous GABA into EVs can significantly suppress seizures. EVs derived from GABAergic interneurons (INs) and medial ganglionic eminence (MGE) cells were particularly effective, while EVs from NPSCs showed limited efficacy [252].

EVs reduce brain damage by suppressing the inflammatory cytokine storm following a seizure. However, it remains unexplored whether EV therapy can reduce the occurrence of seizures. Current studies primarily utilize MSCs as the EV source, with little exploration of other cell sources such as neurons and glia. Additionally, there is a lack of research on the molecular pathways of EVs specific to epilepsy treatment and their effects on electrophysiological properties. With limited investigations on the application of EVs in epilepsy, more studies testing EVs in epileptic models are needed to enhance our understanding of the pathological and therapeutic roles of EVs in this disease.

### Alzheimer’s disease (AD)

In the era of an aging society, AD has been routinely associated with dementia in the elderly [253]. AD pathology manifests as extracellular accumulation of amyloid β (Aβ) plaques and intracellular neurofibrillary tau tangles in cortical and limbic areas [254]. Aβ peptides are phagocytosed by microglia, subsequently triggering immune neuroinflammation. EVs are closely implicated in AD pathogenesis. Microglia spread tau protein through exosomes [85]. Additionally, EVs mediate systemic inflammation and multi-organ dysfunction in AD, such as osteoporosis and cardiovascular diseases [255, 256]. Inhibition of EV biogenesis can reduce Aβ and tau accumulation, subsequently delaying disease progression [257]. Inhibiting ceramide-dependent exosome formation with sphingomyelinase (SMase) silencing improves cognitive function [85, 258, 259]. Similarly, GW4869, an exosome synthesis inhibitor, alleviates neurological deficits in AD [260]. Inhibiting P2X purinoceptor 7 (P2RX7), an ATP-gated cation channel important for microglia’s exosome release, improves memory in animal models [261].

EVs tackle multiple aspects of AD pathogenesis [262]. EVs themselves act therapeutically, functioning as a Trojan horse for Aβ accumulation. Exogenous exosomes markedly reduce Aβ levels in mouse models by binding Aβ to glycosphingolipids (GSLs) and subsequently taken up by microglia [168, 263]. EVs have excellent targeting ability. MSC-EVs are specifically taken up by neurons in pathological regions, suggesting inflammation-driven uptake [264]. However, other studies conversely report that the majority of EVs are internalized by microglia and astrocytes [154]. MSCs are the most popular source of EVs in AD therapy. The proposed mechanisms of MSC-EVs include activation of autophagy through the catalase enzyme, Nrf2 signaling pathway, miR-146a-inhibited NF-κB pathway, and miR-223 targeting the PTEN-PI3K/Akt pathway [155, 156, 159, 265]. Cerebrospinal fluid (CSF) exchange therapy using artificial CSF enriched with MSCs promotes neurogenesis and decreases gliosis in the hippocampus [266]. ASC-EVs similarly decrease Aβ accumulation, neuronal apoptosis, and energy consumption activated by glutamate [161–163], even more effective than MSC-EVs [267]. EVs from other sources, such as NPSCs, neurons, CSF, and human brain microvascular endothelial cells (HBMVECs), also promote neuronal restoration and cognitive recovery [164–169].

Optimized environments affect the therapeutic properties of EVs. A 3D graphene scaffold produces exosomes that more effectively reduce Aβ production [160]. Hypoxia preconditioning decreases pro-inflammatory miR-770-3p and promotes M2 microglia polarization. Similarly, pretreatment with TNF-α and IFN-γ decreases microglia activation and promotes neurite outgrowth [262].

AD is a significant model of neurodegeneration caused by the accumulation of toxic proteins. EV therapy acts as a scavenger for misfolded proteins, an immunomodulator, and a promoter of neurogenesis. The targeting properties of EVs are crucial for precise treatment. However, there are still conflicting reports on the main targets of EVs, and the mechanisms by which EVs target specific cell types or areas of inflammation remain unclear. EVs from different sources may target differently; for example, EVs from astrocytes may have more specificity to neurons than those from MSCs. The extent of EV accumulation in other tissues should also be explored for safety, as EV uptake into normal brain parenchyma may potentially overstimulate proliferation, leading to tumorigenesis. Given the lack of effective treatments for AD, incorporating EV therapy into conventional medication regimens may improve outcomes. Further research is warranted to fully unravel the efficacy and safety profiles and mechanisms underlying EV treatment in AD.

### Parkinson’s disease (PD)

PD corresponds to the second most rampant neurodegenerative disorder behind AD [268, 269]. PD is marked by degeneration of dopaminergic neurons in the substantia nigra within the midbrain. The pathological feature of PD involves the aberrant accrual of α-synuclein, which forms intracellular inclusions known as Lewy bodies. Neuroinflammation plays a crucial role in the pathogenesis of PD [270], and EVs are closely involved in PD pathogenesis [271]. Similar to AD, EVs are implicated in α-synuclein propagation [82]. Leucine-rich repeat serine/threonine kinase 2 (LRRK2), a mutated protein in monogenic PD, is also released through exosomes [83]. Additionally, the prion protein, a glycosylphosphatidylinositol-anchored membrane protein, is involved in α-synuclein transmission through EVs [272, 273].

EVs show potential as a disease-modifying treatment for PD. MSC-derived exosomes can traverse the BBB and exert neuroprotective effects by promoting cell proliferation and inhibiting apoptosis through autophagy induction [170]. Exosomes derived from the dental pulp of human exfoliated deciduous teeth (SHEDs) reduce apoptosis, whereas MVs from the same cells do not provide therapeutic benefits [274]. Exosomes normalize tyrosine hydroxylase expression [171]. Moreover, miR-200a-3p-enriched EVs isolated from healthy astrocytes reduce the expression of mitogen-activated protein kinase kinase 4 (MKK4), a key kinase in the c-Jun N-terminal kinase cell death pathway [172].

PD shares a similar pathogenesis with AD, characterized by the propagation of toxic proteins leading to neuroinflammation. EV therapy can improve PD by promoting neurogenesis. However, the effects of EVs on the primary toxic proteins in PD, such as α-synuclein and LRRK2, are still not fully explored. Blocking exosome release can reduce the propagation of α-synuclein and LRRK2, but the current techniques lack specificity, raising concerns about potential complications. Exosomes and MVs have different effects on PD, with only exosomes exhibiting therapeutic outcomes, emphasizing the importance of EV characterization and selection. Additionally, there is a limited variety of EV sources and modifications in PD therapy compared to other diseases, despite PD being a common cause of dementia. Further modifications of EV sources that cater to targeting the disease hallmark of dopaminergic depletion and degenerative processes (e.g., α-synuclein accumulation) may improve the efficacy of EV-based therapies for PD.

### Amyotrophic lateral sclerosis (ALS)

ALS exhibits a rapid demise of motor neurons in the brain, spinal cord, and peripheral regions, leading to muscle weakness, atrophy, and cognitive impairment [275, 276]. The pathogenesis of ALS involves multiple factors, including genetic mutations in genes like C9orf72, SOD1, TARDBP, and FUS, which result in toxic protein aggregation and impaired RNA processing. Neuroinflammation plays a crucial role, with initial protective responses becoming neurotoxic over time, exacerbated by dysfunctional regulatory T cells (Tregs). Additionally, oxidative stress and mitochondrial dysfunction contribute to cellular damage, while glutamate excitotoxicity further accelerates neuronal death. Despite advancements in understanding these mechanisms, effective therapies remain elusive.

There are only a few studies on EV therapy for ALS [173, 277]. EVs isolated from ASCs can protect SOD1-mutated neurons from oxidative stress and normalize mitochondrial function [175, 176, 278]. MSC-derived EVs were effectively taken up by mouse BECs and restored BBB integrity. MSC-exosomes also promoted neurite growth and upregulated antioxidant and anti-inflammatory genes [173, 174]. In a pilot trial in humans using allogeneic stem cell-derived exosomes, the patient showed signs of stabilization in motor function and respiratory capacity during the infusion period, but deterioration occurred after a pause in treatment [279]. Despite these transient benefits, the patient eventually experienced acute respiratory failure and passed away. Continuous administration may be necessary to maintain benefits.

ALS is a neurodegenerative disorder with a complex pathogenesis. The effects of EV therapy in ALS are primarily focused on reducing oxidative stress and inflammation. However, the sources of EVs are currently limited to MSCs and ASCs. Existing studies are insufficient to fully elucidate the mechanisms of EV therapy in ALS. Pilot studies in humans suggest that intermittent administration of therapeutic EVs may be inadequate for the severe stage. Thus, finding the optimal EV regimen, including dosage and frequency, is needed to enhance therapeutic outcomes of EVs in ALS. Elucidating different EV sources and investigating their specific mechanisms of action may also enhance the efficacy and safety of EV therapy for ALS.

### Multiple sclerosis (MS)

MS displays a chronic inflammation coincident with demyelination of the CNS as evidenced by multifocal zones of inflammatory response, leading to neuronal cell death and nerve demyelination [280, 281]. The pathogenesis of MS involves a complex interplay between genetic predisposition and environmental factors, such as exposure to infectious agents, vitamin deficiencies, and smoking. Inflammation leads to oligodendrocyte death and impaired myelin repair. Oxidative stress driven by microglial activation and mitochondrial injury contributes to demyelination and neurodegeneration. Age-related iron accumulation and mitochondrial gene deletions further amplify these effects, particularly in progressive MS. Although current treatments focus on anti-inflammatory and immunomodulatory drugs, they are insufficient to halt neurodegeneration, necessitating the exploration of novel therapeutic strategies.

EVs play a critical role in the neuroinflammation underlying MS. MSCs are widely used as a source of EVs [282]. EVs isolated from MSCs shift microglial polarization towards the M2 phenotype, increasing Tregs and IL-10 [178, 179]. EVs isolated from human periodontal ligament stem cells suppress inflammation and apoptosis via CD90-inducing IL-10 production [180]. EVs derived from microglia co-cultured with immunosuppressive MSCs promote oligodendrocyte progenitor cell recruitment and differentiation with lipid cargo [181]. Interestingly, EVs released from pro-inflammatory microglia interfere with remyelination only when co-cultured with astrocytes, implying that astrocytes may mediate oligodendrocyte toxicity.

EVs are used as immunomodulation therapy in MS models, primarily sourced from MSCs and microglia. To implement EV therapy more effectively, the molecular mechanisms involved in autoimmune-induced inflammation need further elucidation. EVs from immune cells, such as anti-inflammatory Tregs or M2 microglia/macrophages, may more potently target the pathogenesis of MS. Additionally, EV therapy should focus on other aspects of pathogenesis, such as neuronal degeneration and demyelination. Since MS is a systemic disease, the effects of EVs on other organs should also be explored to ensure comprehensive treatment and safety. By understanding these mechanisms and tailoring the EVs to sequester the complex pathogenesis of MS may reveal the optimal treatment regimen of EVs for this autoimmune disease that compromises nerve cells in both brain and spinal cord.

### Huntington’s disease (HD)

HD is marked by a progressive degeneration of basal ganglia neurons manifesting with behavioral and psychiatric abnormalities [283]. HD is inherited in an autosomal dominant pattern, caused by a mutation in the huntingtin gene (HTT) [284]. An expanded CAG trinucleotide repeats in the HTT gene results in an abnormal huntingtin protein, known as mutant huntingtin (mHTT). The number of CAG repeats directly correlates with the disease’s severity [285]. Accumulation of mHTT in neurons leads to cellular dysfunction, mitochondrial dysfunction, apoptosis, excitotoxicity, and altered gene expression, especially in the striatum and cortex [286]. Similar to other toxic protein propagations, there is evidence that mHTT is transferred by EVs [84, 287–289].

EVs from ASCs and NPSCs effectively restore mitochondrial function, decrease N-terminal cleaved mHTT, and suppress apoptosis [182–184]. EVs isolated from human dermal fibroblasts also recover GABAergic synapses and transmission [185, 186]. Moreover, heterogeneous parasymbiosis in a mouse model showed that blood serum containing therapeutic substances, possibly EVs, can decrease mHTT and neuron degeneration [188]. EVs isolated from human cord blood found that they reduced gliosis, increased antioxidant activity, partially prevented neuronal loss, and effectively improved neuromuscular function [290].

HD is a genetic disorder that currently lacks effective treatments. EV therapy has the potential to reduce mHTT, the main culprit behind neuroinflammation in HD. While there are multiple clinical trials involving cell transplantation for HD, no clinical trials for EV therapy in HD have been conducted yet. Given that mHTT contributes to various aspects of HD pathogenesis, experiments should address multiple domains, including mitochondrial dysfunction, RNA instability, excitotoxicity, and proteolysis impairment. Similar to AD and PD, HD pathogenesis is driven by toxic protein accumulation—in this case, mHTT. Therapeutic EVs have been shown to reduce N-terminal cleavage of mHTT, but whether they can act as scavengers for mHTT remains to be explored. Further research is needed to investigate the direct effects of EVs on HD pathogenesis specifically on reducing mHTT and alleviating its downstream degenerative symptoms.

### Modification and engineering of EVs

Beyond conventional cell-derived EVs, advanced engineering techniques are extensively explored across various stem cell types. In the realm of EV content selection, the biological components of cell-derived EVs can be modulated through culture preconditioning—such as hypoxia or cytokine supplementation—to enhance their therapeutic efficacy, although the resulting outcomes often lack homogeneity [291]. To incorporate small molecules, RNA, and genetic editing tools into EVs, two primary methods of cargo loading are employed: endogenous and exogenous [292–295]. Endogenous loading involves the genetic manipulation of parent cells to ensure that the secreted EVs inherently carry the desired molecules. Conversely, exogenous loading introduces cargo directly onto or into the EV membrane through techniques such as electroporation, ultrasound, extrusion, freeze–thaw cycles, chemical treatments, and mechanical stirring. This method, however, faces challenges related to content volume control, membrane disruption, altered surface electrical charge, and dysfunctional surface ligands, which impair uptake. Targeting EVs represents another critical area of investigation, typically achieved through membrane fusion, chemical modification, and the genetic engineering of membrane peptides [294, 296]. Such modifications enhance fusion efficiency, colloidal stability, and the half-life of EVs in the bloodstream while reducing immunogenicity [297, 298]. Targeting strategies also improve the precision of EV delivery; for instance, Lamp2b-expressing EVs significantly increase uptake in neurons, microglia, and oligodendrocytes within the brain [299]. The engineering of EVs thus emerges as a promising approach for large-scale production and versatile drug delivery platforms. Nonetheless, challenges remain in ensuring reproducibility, safety, and regulatory protocols. With ongoing technological advancements, synthetic EVs hold the potential to become a pivotal component of personalized medicine.

## Conclusion

EVs are crucial mediators of cell-to-cell communication, playing roles in nearly all physiological and pathological processes. EV therapy addresses multiple aspects of neurological diseases, including neuroinflammation, mitochondrial dysfunction, apoptosis, and BBB leakage. Compared to stem cell therapy, EVs are safer and easier to handle, making them a promising alternative for therapeutic interventions.

Despite the rapid growth in the EV field, much remains to be studied. There are numerous EV classes and subclasses yet to be fully characterized. The heterogeneity within EV classes leads to variability in their effects. Studies isolating EVs from the same cell line report different cargo and mechanisms of action. The effects of EV subpopulations are also not fully understood, with research predominantly focused on exosomes. The roles of MVs are still controversial, as they can be either therapeutic or pathogenic depending on their source. MVs and other large EVs may be worth exploring further since they can deliver more cargo. Characterization and purification of EVs are crucial for clinical application. To control the effects of EVs, production methods need to be strictly replicable to avoid heterogeneity, with specific culture and modulation techniques. EVs are involved in multiple pathological processes, such as inflammation, tumorigenesis, and toxic protein spreading. Blocking these pathological EVs is challenging due to a lack of specificity, necessitating more precise techniques for targeting them. Multiple sources of EVs have been studied, but not thoroughly across all domains of treatment. Some cell sources may be better suited for certain roles; for example, stem cells for neuroregeneration, glia for immunomodulation, and endothelial cells for angiogenesis. The targeting ability of EVs is another area that has not been extensively explored. Optimal EV preconditioning, administration regimens, and safety profiles also require further investigation.

A better understanding of EV subtypes and their specific roles will mark a significant milestone in medicine, leading to safer, more effective and disease-tailored EV therapy for a variety of neurological disorders.

## Data Availability

Not applicable.

## Abbreviations

AD: Alzheimer’s disease
AGO2: Argonaute 2
ALS: Amyotrophic lateral sclerosis
ApoD: Apo-lipoprotein D
AQP-4: Aquaporin-4
ARMMs: Arrestin domain-containing protein 1-mediated microvesicles
ARRDC1: Arrestin domain-containing protein 1
ASCs: Adipose-derived stem cells
Aβ: Amyloid β
BDNF: Brain-derived neurotrophic factor
BBB: Blood–brain barrier
BECs: Brain endothelial cells
Cav-1: Caveolin-1
CK18: Cytokeratin 18
CNS: Central nervous system
COX-2: Cyclooxygenase-2
CSF: Cerebrospinal fluid
ESCRT: Endosomal sorting complexes required for transport
EVs: Extracellular vesicles
eef2k: Eukaryotic elongation factor 2 kinase
FGF-1: Fibroblast growth factor 1
GDNF: Glial cell line-derived neurotrophic factor
GSLs: Glycosphingolipids
HBMVECs: Human brain microvascular endothelial cells
HD: Huntington’s disease
HIF-1: Hypoxia-inducible factor-1
HTT: Huntingtin gene
IFN: Interferon
IGF-1: Insulin-like growth factor 1
IL: Interleukin
ILVs: Intraluminal vesicles
INs: Interneurons
iPSCs: Induced pluripotent stem cells
I-BAR: Bin–Amphiphysin–Rvs
LCN2: Lipocalin 2
LRRK2: Leucine-rich repeat serine/threonine kinase 2
MGE: Medial ganglionic eminence
MALAT1: Metastasis-associated lung adenocarcinoma transcript 1
miRNA: MicroRNA
MKK4: Mitogen-activated protein kinase kinase 4
MVBs: Multivesicular bodies
MVs: Microvesicles
mHTT: Mutant huntingtin
MSCs: Mesenchymal stem cells
MS: Multiple sclerosis
NET: Neutrophil extracellular trap
NGF: Nerve growth factor
NMDA: *N*-Methyl-d-aspartate
NPSCs: Neural stem cell/progenitor cell
NSCs: Neural stem cells
OECs: Olfactory ensheathing cells
OPC: Oligodendrocyte progenitor cell
PD: Parkinson’s disease
P2RX7: P2X purinoceptor 7
PDCD6IP (also ALIX): Programmed cell death 6-interacting protein
PNI: Peripheral nerve injury
PNS: Peripheral nervous system
ROS: Reactive oxygen species
SCs: Schwann cells
SCI: Spinal cord injury
SE: Status epilepticus
SHEDs: Dental pulp of human exfoliated deciduous teeth
SRSF2: Splice factor serine-arginine-rich splice factor 2
STAU1: Staufen homolog 1
TBI: Traumatic brain injury
TLR4: Toll-like receptor-4
TNSC6A (also GW182): Trinucleotide repeat-containing gene 6A protein
TNF-α: Tumor necrosis factor alpha
Tregs: Regulatory T cells
TSG101: Tumor susceptibility gene 101 protein
TSPAN4: Tetraspanin-4
TRPM7: Transient receptor potential melastatin 7
VEGF: Vascular endothelial growth factor
